# HADHA promotes esophageal cancer progression by activating mTOR signaling and the SP1/MDM2 axis: HADHA promotes esophageal cancer progression

**DOI:** 10.3724/abbs.2024139

**Published:** 2024-09-26

**Authors:** Xusheng Ding, Longlong Shao, Jie Wang, Yongwei Jin, Haiqing Chen, Bin Li

**Affiliations:** 1 Departments of Thoracic Surgery and State Key Laboratory of Genetic Engineering Fudan University Shanghai Cancer Center Shanghai 200032 China; 2 Institute of Thoracic Oncology Fudan University Shanghai 200032 China; 3 Department of Oncology Shanghai Medical College Fudan University Shanghai 200032 China; 4 Department of Thoracic Surgery Fudan University Shanghai Cancer Center Xiamen Hospital Xiamen 361026 China

**Keywords:** HADHA, esophageal cancer, mTOR signaling, MDM2, SP1

## Abstract

Esophageal cancer (EC) is one of the most recalcitrant cancers, with a 5-year survival rate of < 30%. The hydroxyacyl-CoA dehydrogenase alpha subunit (HADHA) plays an essential role in long-chain fatty acid metabolism, and dysregulation of HADHA has been demonstrated to be involved in a series of metabolic diseases and cancers. However, its role in cancers remains controversial. HADHA has seldom been investigated in EC, and little is known about how HADHA regulates the malignant progression of EC. In this study, we find that HADHA is significantly upregulated in EC tissues and is correlated with poor survival. *HADHA* knockdown markedly inhibits EC cell proliferation both *in vitro* and *in vivo*. The loss of HADHA also induces EC cell apoptosis, causes cell cycle arrest and inhibits cell migration. Additionally, RNA profiling reveals that mTOR signaling is significantly suppressed after *HADHA* knockdown. Mechanistically, HADHA interacts with SP1 and induces MDM2 expression. In conclusion, both mTOR signaling and the SP1-MDM2 axis participate in the HADHA-induced malignant behavior of EC cells.

## Introduction

Esophageal cancer (EC) is one of the most lethal cancers, with a 5-year relative survival rate of only 20%‒30% [1]. Even if it is detected in the early stages, the 5-year relative survival rate is still less than 50%, and this rate decreases to only 5% in the metastatic stage [2]. Globally, it is estimated that EC is the ninth most common cause of newly diagnosed tumors and ranks sixth in mortality. Additionally, the incidence of EC differs among geographic distributions, with Eastern Asians having the highest incidence in both men and women, partly because of the heavy burden in China [3]. Surgery remains the main strategy for the treatment of early-stage EC [4]. For localized and regional EC, trimodality (chemoradiation followed by surgery) has emerged as the standard treatment strategy, whereas advanced EC largely relies on traditional chemotherapy with immunotherapy (anti-PD-L1 agents) [5]. Current therapeutic advancements are limited, and new targets need to be further investigated.

The hydroxyacyl-CoA dehydrogenase alpha subunit (HADHA) is one of the two subunits of the mitochondrial trifunctional protein, which is responsible for the last three steps of the mitochondrial beta-oxidation of long-chain fatty acids [6]. Loss of HADHA expression results in the accumulation of long-chain fatty acid metabolites [7]. Accordingly, HADHA is essential for regulating energy metabolism and maintaining metabolic homeostasis, and HADHA dysfunction is involved in the pathogenesis of multiple human diseases, including metabolic disorders, cardiovascular diseases, and inflammatory bowel disease [ 8‒ 10]. Recently, HADHA was reported to play an important role in tumorigenesis. In lung cancer and lymphoma, HADHA acts as an oncogene by promoting tumor cell proliferation and decreasing susceptibility to chemotherapy reagents [ 11, 12]. In contrast, HADHA overexpression retards tumor growth in clear cell renal cell carcinoma and is correlated with favorable patient survival [13]. However, the role of HADHA in EC has rarely been discussed, and investigating its functional status could identify potential therapeutic targets for EC treatment.

In the present study, we demonstrated that HADHA was upregulated in EC tissues and patients with high HADHA expression had poorer survival. Overexpression of HADHA resulted in enhanced proliferation and migration abilities of EC cells *in vitro* and *in vivo*. Functional studies showed that both mTOR inhibitor and downregulation of MDM2 abrogated HADHA-induced EC cells progression. Taken together, these results indicated that HADHA is a new oncogene related with EC.

## Materials and Methods

### Patient samples, gene expression profiling and immunohistochemical (IHC) staining

Clinical EC tissue samples were obtained from 389 patients who underwent operations at Fudan University Shanghai Cancer Center (FUSCC). All tissue samples were pathologically confirmed as ECs. Patients were followed up every 3 months for two years after surgery and then every 6 months until death. The expression of HADHA was evaluated via IHC staining with an anti-HADHA antibody (ab203114; Abcam, Cambridge, USA), according to standard IHC procedures. Anti-HADHA antibody was used at a dilution of 1:250. The protein expression level was evaluated and scored by two pathologists independently on the basis of the positive area and intensity. The positive area scores were determined as follows: 0, no positive area according to IHC staining; 1, 0%‒25%; 2, 25%‒50%; 3, 50%‒75%; and 4, ≥ 75%. The intensity scores of IHC were divided into 0‒3; no positivity in the cytoplasm, membrane or nucleus was considered a score of 0, and strong positivity as a brown color was considered a score of 3. The intensity score was multiplied by the positive area score to generate the final IHC result: 0, negative; 1‒12, positive (1‒4, +; 5‒8, ++; and 9‒12, +++). This study was approved by the Ethics Committee of Fudan University Shanghai Cancer Center.

### Cell lines

The human EC cell lines EC9076, KYSE450, Eca-109 and TE-1 were obtained from the American Type Culture Collection (ATCC; Manassas, USA). All the cell lines were maintained in RPMI 1640 medium supplemented with 10% fetal bovine serum (Gibco®; Thermo Fisher Scientific, Waltham, USA) and incubated at 37°C in an atmosphere of 5% CO _2_.

### Western blot analysis

After being collected and washed with phosphate-buffered saline (PBS), the cells were lysed on ice for 30 min in RIPA buffer containing a protease inhibitor cocktail (Cell Signaling Technology, Beverly, USA). The protein concentration was determined using a BCA protein assay reagent kit (Beyotime, Nanjing, China). A total of 20 μg of protein was loaded onto 10% SDS-polyacrylamide gels for electrophoresis. The proteins were then transferred to PVDF membranes (Beyotime). After being blocked with 5% nonfat milk, the membranes were incubated overnight with primary antibodies specific against HADAH (ab203114, 1:1000 dilution; Abcam), MDM2 (ab259265, 1:1000 dilution; Abcam), p-mTOR (ab109268, 1:2000 dilution; Abcam), mTOR (ab32028, 1:1000 dilution; Abcam), PPP2CA (ab32141, 1:5000 dilution; Abcam), PIK3R1 (30092-1-AP, 1:2000 dilution; Proteintech, Shanghai, China), PIK3CB (67121-1-Ig, 1:5000 dilution; Proteintech), IRS1 (17509-1-AP, 1:1000 dilution; Proteintech), SP1 (21962-1-AP, 1:500 dilution; Proteintech), GAPDH (60004-1-lg, 1:10000 dilution; Proteintech). Next, the membranes were washed three times and incubated with HRP-conjugated anti-rabbit IgG (A0208, 1:1000; Beyotime) or anti-mouse IgG (A0216, 1:1000; Beyotime) secondary antibody for 2 h at room temperature. Finally, a chemiluminescent Western Blotting Substrate (Thermo Fisher Scientific) and infrared imaging system (LI-COR Biosciences, Lincoln, USA) were used to visualize the protein bands. GAPDH was used as the loading control.

### RNA extraction and quantitative PCR

Trizol (#T9424-100M; Sigma, St Louis, USA) was used to extract total RNA, and Hiscript QRT Supermix for qPCR (#R123-01; Vazyme, Nanjing, China) was used for reverse transcription. For quantitative PCR analysis, a 20-μL volume was used with 2 μL cDNA, 0.5 μM specific primers and 10 μL SYBR Green Mix (11184ES08; Yeasen Biotechnology, Shanghai, China), supplemented to 20 μL with ddH _2_O. The amplification conditions were 95°C for 30 s, and then 40 cycles of 95°C for 3 s and 60°C for 20 s. The expressions of specific genes were detected via real-time PCR using an ABI 7900HT real-time PCR system (Applied Biosystems, Foster City, USA). *GAPDH* was used as an internal control. The primer sequences of all the genes are listed in Supplementary Table S1.

### Plasmids and transfection

To silence target gene expression, the BR-V108 cloning vector (Shanghai Biosciences Co. Ltd., Shanghai, China) was used. The 22 bp target of *HADHA* was 5′-ATGCTGACTGGTAGAAGCATT-3′. The 21-bp target of murine double minute 2 ( *MDM2*) was 5′-AGGGAAGAAACCCAAGACAAA-3′. The target sequence of 5′-TTCTCCGAACGTGTCACGT-3′ was used as a control scrambled shRNA, according to previous research [14]. The Ubi-MCS-3FLAG-CBhgcGFP-IRES-puromycin vector was used to overexpress HADHA, and an empty vector was used as a control. Coinfection of the vector with psPAX2 and pMD2G (4:3:2) into 293T cells produced a lentivirus. Two days after transfection, the recombinant lentiviral virus was collected and used to infect Eca-109 and TE-1 cells, and fluorescence was used to evaluate the degree of infection efficiency.

### Celigo cell counting assay

For the cell counting assay, approximately 2000 Eca-109 and TE-1 cells were seeded in three replicates on day zero in 96-well plates. After 24 h of plating, a Celigo Imaging Cytometer (Nexcelom Bioscience, Boston, USA) was used every 24 h until day six to scan the cells and obtain images. The cell numbers are presented as the fold change in the number of cells in each group on day one.

### Wound healing assay

Eca-109 and TE-1 cells were seeded into 96-well plates, and 90% confluent cells were used for the wound healing assay. A 96 Wounding Replicator (V&P Scientific, San Diego, USA) was used to scratch the cell monolayers, and PBS was used to wash the monolayers twice. The cells were then cultured in serum-free medium. The wound width was measured and analyzed at 0 and 48 h via Cellomics (Thermo Fisher Scientific). A marker was placed at the bottom of the plate to determine the position of the captured wounds.

### Transwell assay

Eca-109 and TE-1 cells (approximately 150,000 cells) were seeded in the upper surface of transwell chambers (Corning, Shanghai, China) and cultured in 100 μL of serum-free medium. A total of 600 μL of medium supplemented with 30% FBS was added to the plates. After 24 h, the cells in the Transwell chambers were washed and fixed. The lower surface of the chambers was imaged, and the number of cells was counted.

### Flow cytometry analysis

Apoptosis was detected via an Annexin V Apoptosis Kit-PE (Southern Biotech, Birmingham, USA) according to the manufacturer’s instructions. Cell cycle analysis was performed via the use of a propidium iodide cell cycle reagent (P4170; Sigma), and the samples were analyzed using a Guava easyCyte flow cytometer (Millipore, Billerica, USA).

### RNA sequencing and bioinformatics analysis

The Eca-109 cells stably transfected with shNC or shHADHA were used to identify differentially expressed mRNAs. RNA was tracted from the three samples of each group using Trizol (#T9424-100M; Sigma) and sent to Tongyuan Medical Laboratory Co., Ltd (Nanjing, China) for mRNA sequencing. Illumina TruSeq ^TM^ RNA sample prep kit (Illumina Inc., San Diego, USA) was used to create the libraries. Differentially expressed genes were defined as |logFC| ≥ 1.3 and FDR<0.05. The volcano plots and heat maps were analyzed and visualized using R/Bioconductor packages ggplot2 and pheatmap. Ingenuity pathway analysis (IPA) was used to perform enrichment analysis of pathways and predict downstream targets. The TRRUST database ( https://www.grnpedia.org/trrust) was used to predict the upstream transcription factors of MDM2. The STRING database ( https://cn.string-db.org) was used to identify potential proteins that interact with HADHA.

### Promoter activity with dual luciferase assay

The *MDM2* promoter region covering from ‒2000 to +300 was amplified and cloned and inserted into the GL002-pGL-luciferase-SV40poly(A) vector (Promega, Madison, USA). JASPAR ( http://jaspar.genereg.net) was used to predict the binding sites of SP1 in the *MDM2* promoter, and a mutated GL002 with a mutated *MDM2* promoter was constructed as a mut control. Each experiment was repeated three times, and luciferase activity was examined using the Dual-Luciferase Reporter Assay System (Promega).

### Co-immunoprecipitation (Co-IP)

Co-IP was performed with anti-HADHA antibody (1:400; Abcam). Cell lysis buffer containing a protease inhibitor cocktail was used to extract whole-cell lysates. The supernatant, which was collected from centrifugation, was incubated with anti-HADHA antibody (1:1000 dilution) overnight at 4°C. Subsequently, protein A/G beads were added to the supernatant and incubated for 2 h at room temperature. Beads were washed with cold lysis buffer, mixed with 2× SDS buffer, heated, and then subjected to western blot analysis.

### Chromatin immunoprecipitation (ChIP)

A ChIP assay was used to determine the binding of the SP1 and *MDM2* promoters following the protocol of the SimpleChIP® Enzymatic Chromatin IP Kit (#9002; Cell Signaling Technology). The *MDM2* promoter region primers used were as follows: forward primer, 5′-CTTCTGAGATGGAGTCTTGCTCTG-3′; and reverse primer, 5′-CGCCTGTAATCCTAGCCACTTG-3′. The crosslinked protein/DNA complex was immunoprecipitated with an anti-SP1 antibody and isotype control IgG, followed by incubation with ChIP-grade protein G agarose beads for 2 h at 4°C. The final amplified targeted DNA sequence was resolved on agarose gels for ChIP experiments.

### *In vivo* experiments

Four-week-old healthy male athymic nude mice were purchased from the Institute of Zoology, Chinese Academy of Sciences (Beijing, China). The mice were randomly divided into two groups ( *n* = 5), and HADHA shRNA-expressing and control Eca-109 cells (2 × 10 ^6^) were subcutaneously injected into the nude mice. One week after injection, the tumor volume was measured every week. All the mice were sacrificed after five weeks, and the neoplasms were excised for IHC. All animal experiment procedures were performed in accordance with the official recommendations of the Chinese Animal Community and approved by the FUSCC Ethics Committee.

### Statistical analysis

All the statistical analyses were performed via SPSS (version 24.0; IBM Inc., Armonk, USA) or GraphPad Prism (version 8; GraphPad Software, La Jolla, USA). Continuous data from the two groups were analyzed using the independent Student’s *t* test or Wilcoxon signed rank test. One-way analysis of variance was used to analyze continuous data from three or more groups. A Kaplan-Meier plotter was used to examine the survival correlations. Statistical significance was set at *P* < 0.05.

## Results

### HADHA upregulation was identified in EC and correlated with poor survival

**Figure 1:**
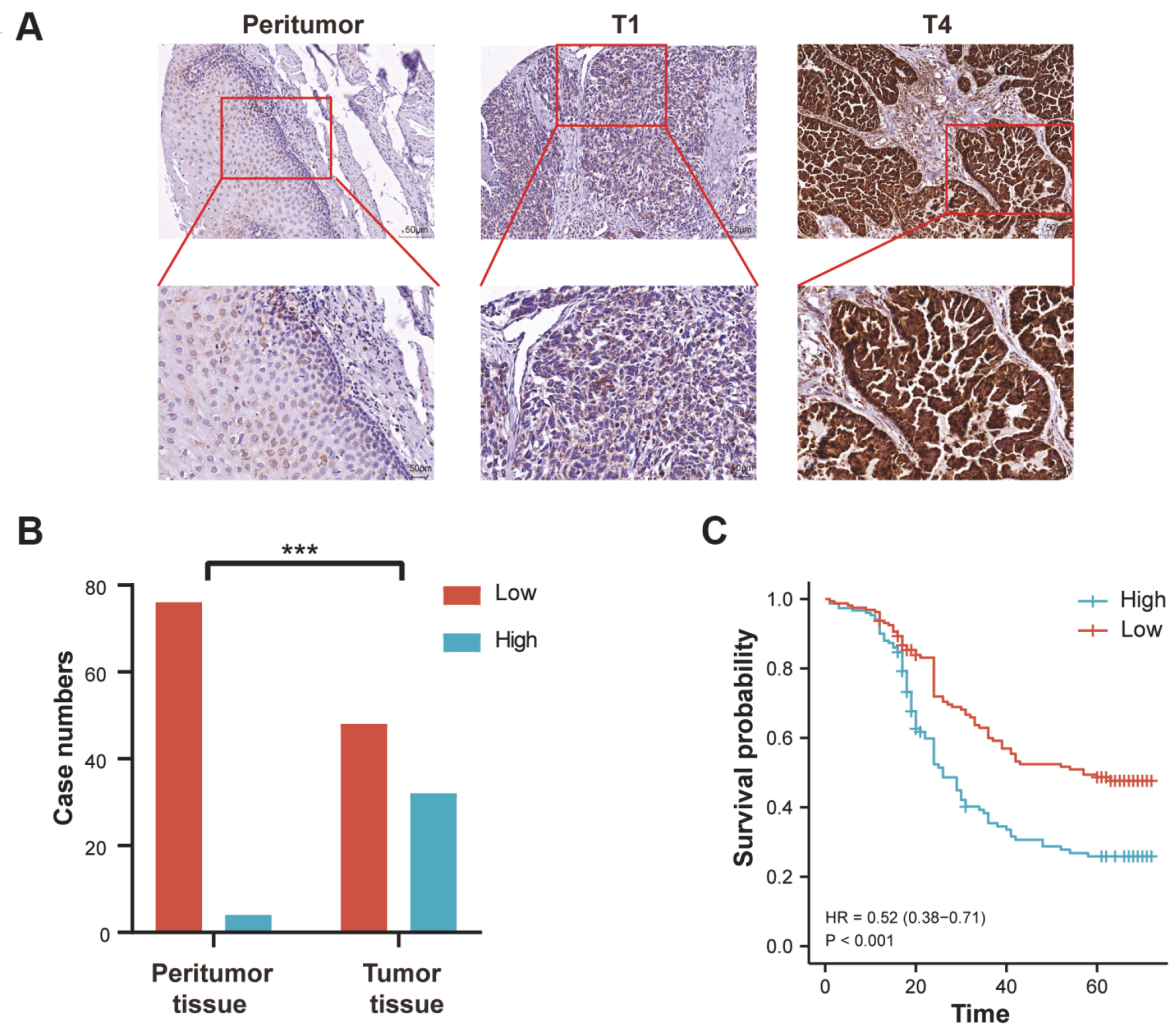
Figure 1 HADHA expression in tissues and was correlated with poor survival (A) Expression of HADHA was examined by immunohistochemistry in peritumor and EC tissues. (B) Diagram of the expression of HADHA in 80 paired EC tissues. (C) Kaplan-Meier curves of overall survival of patients with EC according to HADHA expression. ***P < 0.001.

Since the expression of HADHA in EC has never been evaluated, we first performed IHC staining of EC tissue samples from different tumor stages. Compared with peritumor tissues, EC tissues exhibited significantly higher positivity for HADHA ( Figure 1A). In a group of 80 paired samples from patients with EC, HADHA expression was significantly higher in tumor tissues than in peritumoral tissues ( *P* < 0.001; Figure 1B). To further confirm the significance of HADHA expression in EC, we investigated its correlation with the pathological characteristics and survival of 389 patients. High HADHA expression was significantly related to tumor infiltration, lymphatic metastasis, tumor stage, and distant metastasis, indicating more aggressive tumor behavior ( Table 1). Additionally, patients with high HADHA expression had poorer survival, with a median survival time of 24 months versus 36 months (hazard ratio, 0.52; 95% confidence interval, 0.38–0.71; *P* < 0.001) ( Figure 1C).

**Table:** **Table 1** Correlation of HADHA expression with clinicopathologic features

Features	Number of patients	HADHA expression		*P* value
		Low	High		
All patients	389	195	194		
Sex					0.140
Male	309	149	160		
Female	80	46	34		
T infiltrate					0.000***
T0	7	7	0		
T1	30	26	4		
T2	94	52	42		
T3	218	98	120		
T4	20	8	12		
Lymphatic metastasis (N)					0.000***
N0	200	122	78		
N1	92	37	55		
N2	57	23	34		
N3	20	9	11		
Stage					0.000***
0	6	6	0		
I	82	60	22		
II	124	56	68		
III	144	60	84		
IV	13	9	4		
Lymphoid positive number					0.694
≤ 1	43	15	28		
> 1	39	12	27		
Metastasis (M)					0.024*
0	358	182	176		
1	9	8	1		
Grade					0.341
I	7	2	5		
II	46	15	31		
III	23	10	13

### *HADHA* knockdown suppressed EC proliferation *in vitro* and *in vivo*

**Figure 2:**
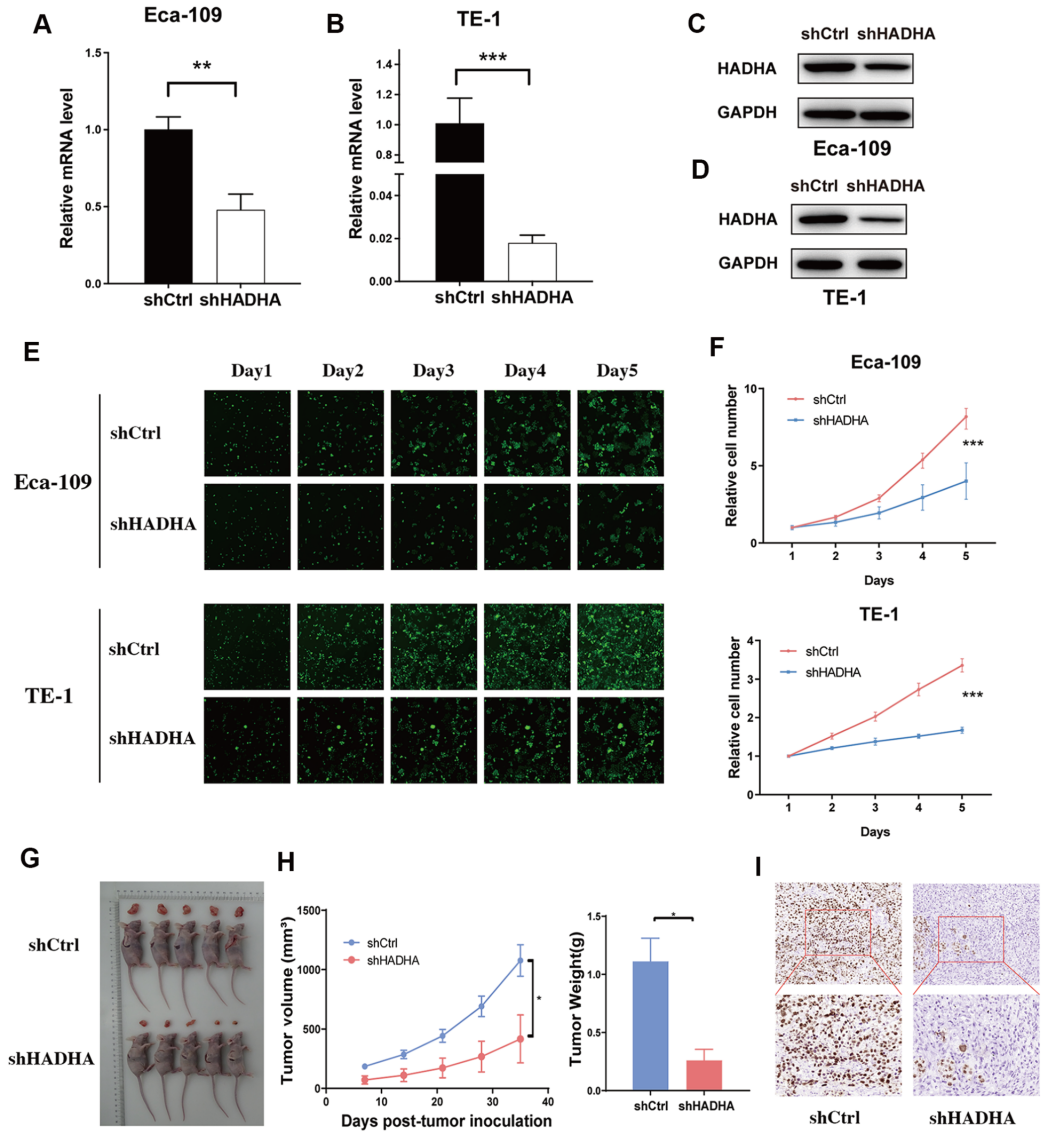
Figure 2 *HADHA* knockdown suppressed EC proliferation *in vitro* and *in vivo* (A,B) Relative mRNA expression of HADHA was examined in Eca-109/Ctrl and Eca-109/shHADHA cells (A), and TE-1/Ctrl and TE-1/shHADHA cells (B). (C,D) Stable reduction of HADHA was confirmed by western blot analysis in Eca-109 (C) and TE-1 cells (D). (E) Celigo cell counting assay was performed to assess the abilities of EC cell growth. Magnification: 100×. (F) Cell proliferation curves of EC cells were measured by Celigo cell counting. (G) Representative photographs of nude mice tumors derived from Eca-109/shCtrl and Eca-109/shHADHA cells. (H) Growth kinetics curves and average tumor weigh of tumors in nude mice implanted with Eca-109/shCtrl and Eca-109/shHADHA cells. (I) Expression of Ki67 was examined by immunohistochemistry in tumors from Eca-109/shCtrl and Eca-109/shHADHA groups. *P < 0.05, **P < 0.01, ***P < 0.001.

To evaluate the role of HADHA in EC cells, the mRNA levels o *f HADHA* in four EC cell lines were examined ( Supplementary Figure S1A). Compared with EC9076 and KYSE450 cells, Eca-109 and TE-1 cells presented higher levels of *HADHA* mRNA. HADHA-specific shRNA lentiviruses were used to abrogate HADHA expression in Eca-109 and TE-1 cells. The knockdown efficacy was confirmed at the mRNA and protein levels ( Figure 2A‒D and Supplementary Figure S1B‒F). Since HADHA reportedly modulates cell proliferation in different tumors, we performed a Celigo cell counting assay to examine the effect of *HADHA* knockdown on EC cells. In both Eca-109 and TE-1 cells, the loss of *HADHA* expression significantly inhibited EC cell proliferation ( Figure 2E,F). We examined tumor formation *in vivo* and found that *HADHA* knockdown significantly abrogated the proliferation of Eca-109 cells ( Figure 2G,H). Additionally, Ki-67 expression in mouse xenografts was assessed by IHC staining, and the results revealed that HADHA expression was positively correlated with Ki-67 expression ( Figure 2I).

### *HADHA* knockdown induced EC cell apoptosis and inhibited cell migration

**Figure 3:**
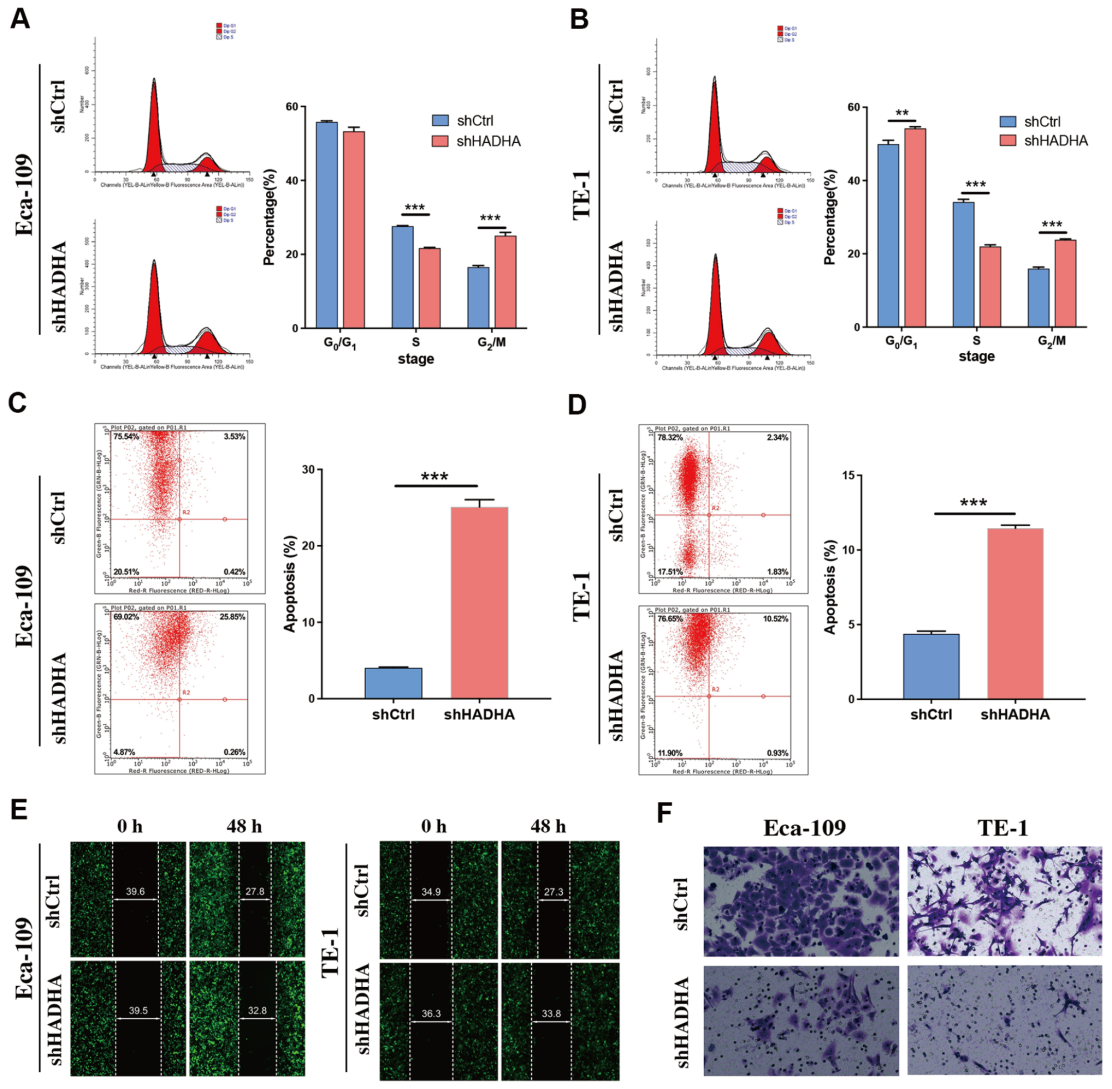
Figure 3 Effect of HADHA on the cell cycle, apoptosis, and migration in EC cells (A,B) HADHA knockdown in Eca-109 (A) and TE-1 (B) cells led to a decrease of cells at S phase and increase of cells at G2/M phase; the statistical results are shown on the right. (C,D) HADHA knockdown in Eca-109 (C) and TE-1 (D) cells increased apoptosis. The statistical results are shown on the right. (E) HADHA knockdown suppressed EC cells migration in wound healing assay. Magnification: 100×. (F) HADHA knockdown suppressed EC cells migration in transwell assay. Magnification: 200×. **P < 0.01, ***P < 0.001.

Since HADHA promoted EC growth, we investigated its effect on the cell cycle. As shown in Figure 3A,B, the loss of *HADHA* expression led to a significant decrease in the proportion of tumor cells in the S phase and a significant increase in the proportion of those in the G _2_/M phase. We subsequently examined the level of apoptosis in separate groups and found that *HADHA* knockdown markedly increased the apoptotic rates of Eca-109 and TE-1 cells ( Figure 3C,D). Additionally, in the wound healing assay, the loss of *HADHA* suppressed cell migration ( Figure 3E and Supplementary Figure S1G). Similar results were observed in the transwell migration assay ( Figure 3F and Supplementary Figure S1H).

### RNA expression profiling identified mTOR signaling and MDM2 as HADHA downstream targets

**Figure 4:**
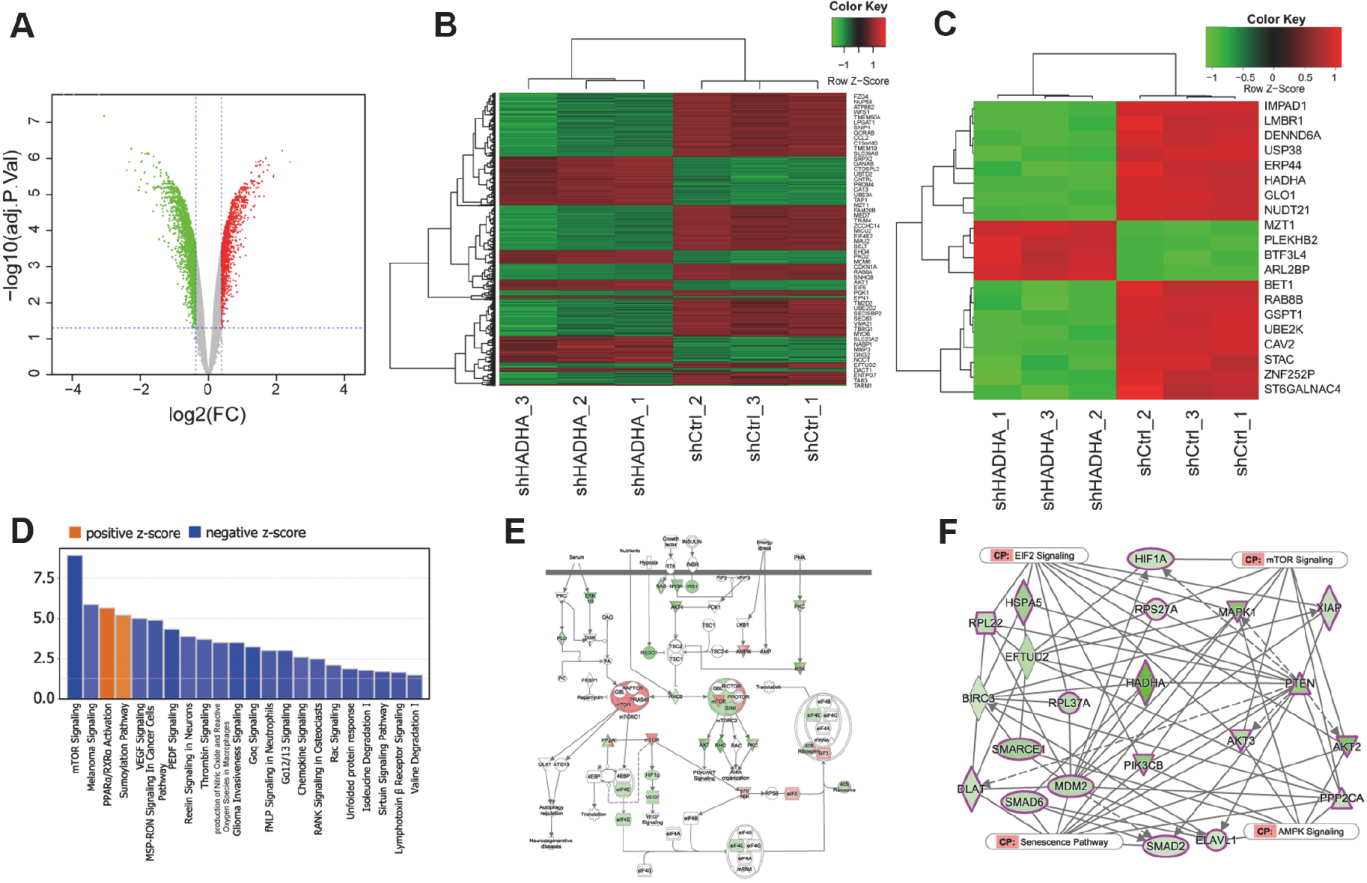
Figure 4 RNA expression profiling identified mTOR signaling and MDM2 as HADHA downstream targets (A,B) Volcano plot (A) and heatmap (B) were used to visualize differentially expressed genes between Eca-109/shCtrl and Eca-109/shHADHA cells. (C) A heatmap was used to visualize the top 20 differentially expressed genes. (D) IPA-based Pathway Analysis identified key pathways affected by HADHA knockdown. (E) IPA Pathway Prediction Analysis showed genes downregulated by HADHA knockdown EC cells. (F) IPA-generated Graphical Summary to predict downstream targets.

The traditional function of HADHA is to metabolize long-chain fatty acids; however, its role in tumorigenesis has seldom been investigated. To further explore the molecular mechanism by which HADHA facilitates tumor malignancy, we carried out RNA sequencing of HADHA-shRNA-transfected and control Eca-109 cells. A total of 792 upregulated genes and 1366 downregulated genes were identified on the basis of the criteria of > ±1.3-fold changes and FDR < 0.05 ( Figure 4A,B). These differentially expressed genes clearly distinguished HADHA-shRNA-transfected Eca-109 cells from scrambled shRNA-transfected Eca-109 cells. The top 20 DEGs are shown in Figure 4C. Enrichment analysis was subsequently performed on the DEGs via ingenuity pathway analysis (IPA). Interestingly, IPA revealed that mTOR signaling was the top upstream regulator, with a Z score of ‒3.162 ( Figure 4D). Additionally, the mTOR signaling pathway was significantly inhibited, and a pair of participating genes was downregulated in HADHA-deficient EC cells ( Figure 4E). Furthermore, we explored HAHDA-related genes in four traditional signaling pathways, including the mTOR signaling pathway, to predict downstream targets using IPA software and found that MDM2 could be a potential direct target of HADHA ( Figure 4F). In conclusion, the proliferative function of HADHA might be largely attributed to the mTOR signaling pathway.

### HADHA promoted EC progression through mTOR signaling

**Figure 5:**
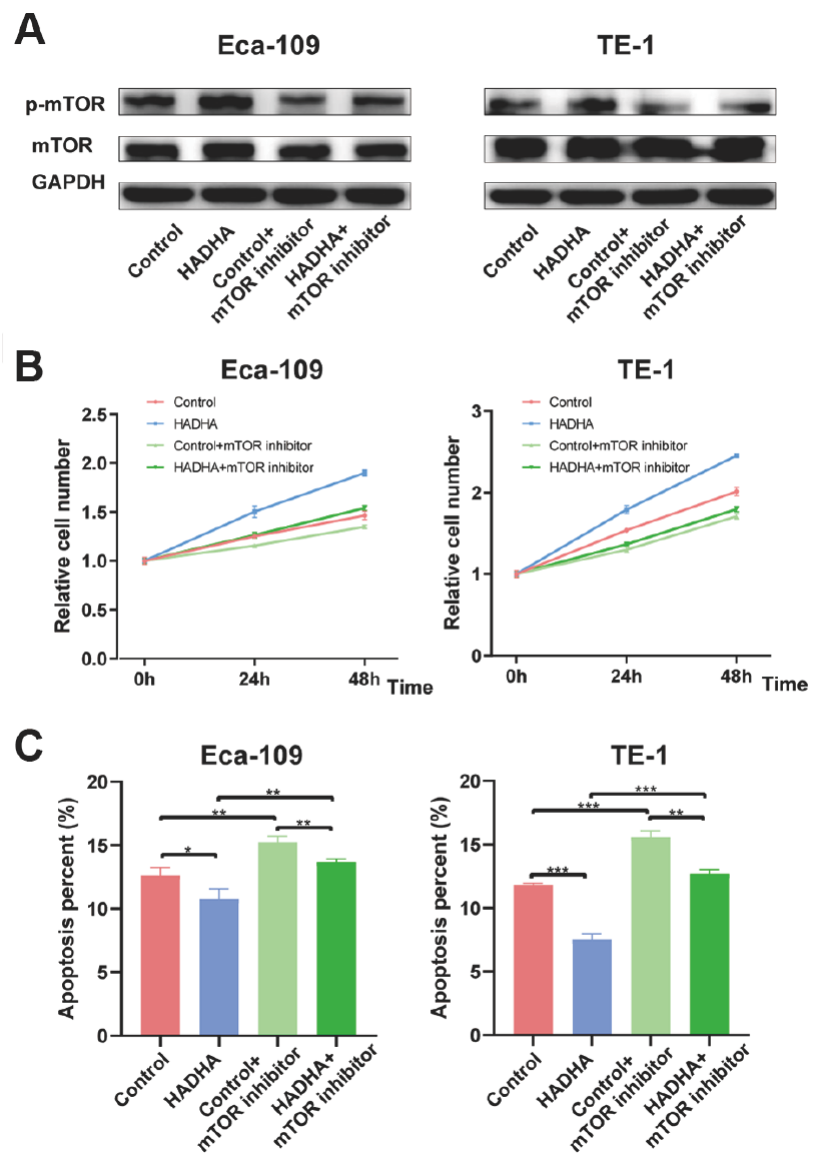
Figure 5 HADHA promoted EC progression through mTOR signaling (A) HADHA-induced mTOR signaling activation was confirmed by western blot analysis in EC cells. (B) HADHA promoted EC cells proliferation through mTOR signaling. (C) HADHA inhibited EC cells apoptosis through mTOR signaling. *P < 0.05, **P < 0.01, ***P < 0.001.

To examine the effect of HADHA on the mTOR signaling pathway, western blot analysis was used to assess the mTOR and p-mTOR levels. Upregulation of HADHA markedly increased p-mTOR level, which was reversed by rapamycin (an mTOR inhibitor) ( Figure 5A). In addition, rapamycin blocked the effect of HADHA on cell proliferation ( Figure 5B). HADHA overexpression inhibited apoptosis, whereas rapamycin markedly reversed this effect ( Figure 5C). These results showed that HADHA promoted EC progression by activating the mTOR signaling pathway.

### HADHA promoted EC progression by upregulating MDM2 expression

**Figure 6:**
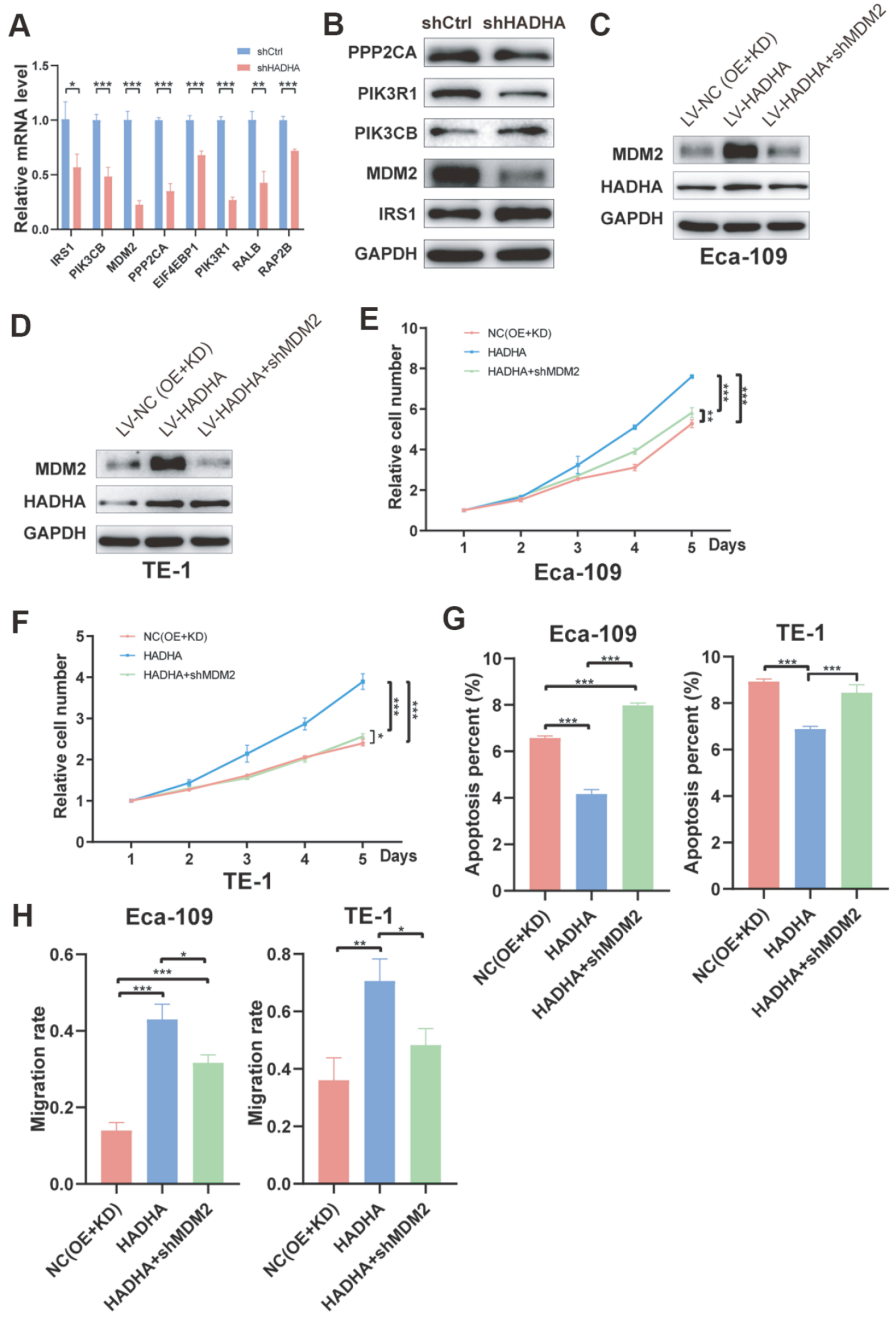
Figure 6 HADHA promoted EC progression by upregulating MDM2 expression (A,B) The relative mRNA expression (A) and protein levels (B) of differentially expressed genes were examined. (C,D) HADHA upregulated MDM2 expression in Eca-109 (C) and TE-1 (D) cells. (E,F) HADHA promoted EC cells proliferation by upregulating MDM2 expression in Eca-109 (E) and TE-1 (F) cells. (G,H) HADHA inhibited EC cells apoptosis (G) and promoted EC cells migration (H) by upregulating MDM2 expression. *P < 0.05, **P < 0.01, *** P < 0.001.

To determine the specific downstream targets of HADHA in EC, we evaluated several differentially expressed genes, including *IRS1*, *PIK3CB* , *MDM2*, *PPP2CA*, *EIF4EBP1*, *PIK3R1*, *RALB* and *RAP2B*, at both the mRNA and protein levels. The mRNA and protein levels of all these genes were significantly downregulated in *HADHA*-silenced cells, with MDM2 protein expression being the most obvious ( Figure 6A,B). MDM2 is a traditional oncogenic protein that blocks the transcriptional activation domain of p53 [15]. To further confirm the role of MDM2 in EC, HADHA was upregulated, and MDM2 was downregulated in EC cells ( Figure 6C,D and Supplementary Figure S2A,B). In the following functional assays, we found that HADHA markedly promoted the proliferation of Eca-109 and TE-1 cells, which was reversed by *MDM2* knockdown ( Figure 6E,F and Supplementary Figure S2C). In addition, HADHA significantly inhibited the apoptosis of Eca-109 and TE-1 cells, which was reversed by *MDM2* knockdown ( Figure 6G and Supplementary Figure S2D). Furthermore, silencing of *MDM2* reversed the increase in cell migration ability induced by HADHA ( Figure 6H). These results indicated that HADHA promoted EC progression by upregulating MDM2 expression.

### HADHA interacted with SP1 to promote MDM2 expression

**Figure 7:**
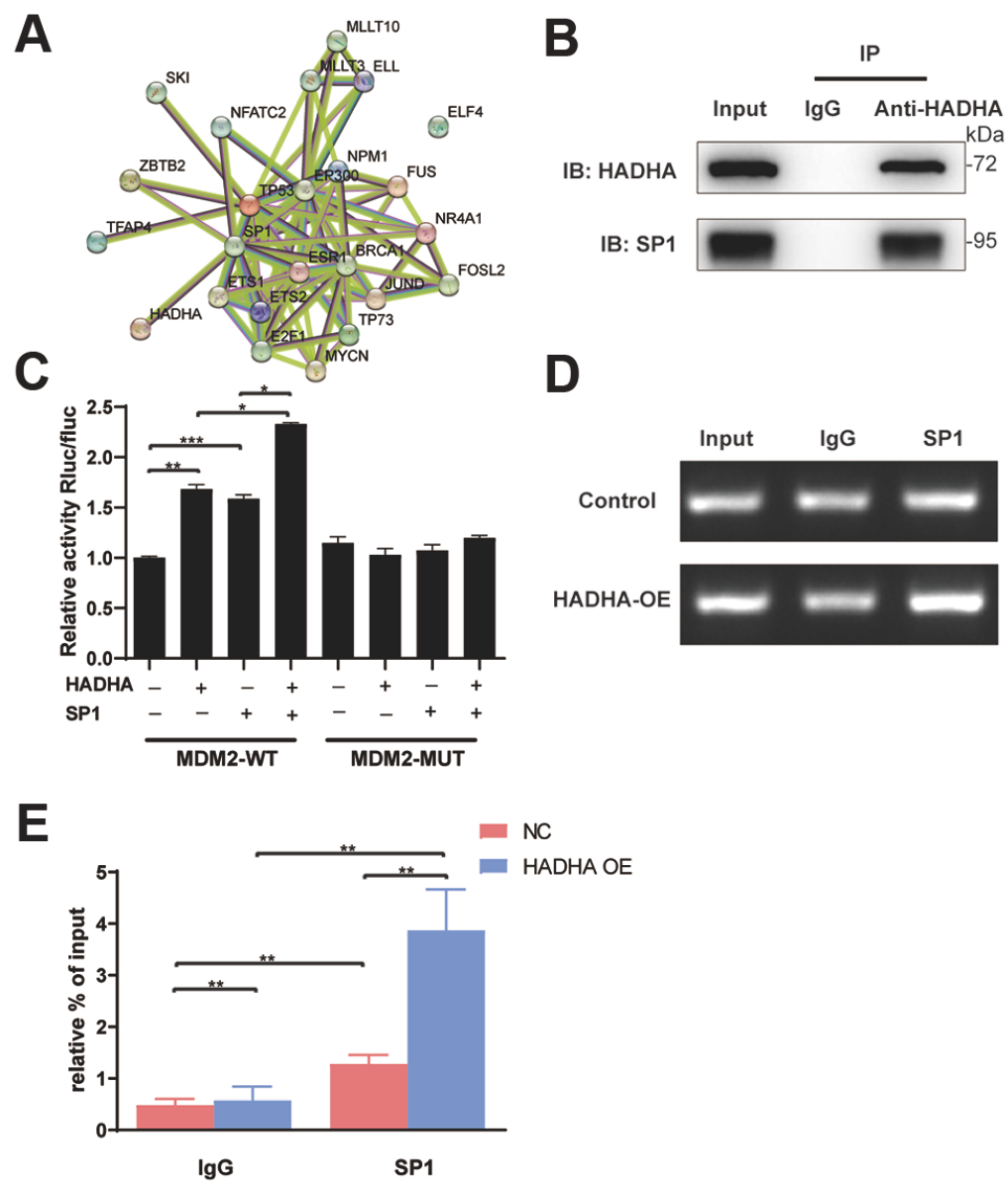
Figure 7 HADHA interacted with SP1 to promote MDM2 expression (A) STRING gene interaction network was generated to identify potential interacting proteins. (B) Co-immunoprecipitated was performed to identify the interaction between HADHA and SP1. (C) The dual-luciferase reporter assay was performed, and relative luciferase activity was detected, respectively. (D,E) ChIP analysis was performed in Eca-109/Ctrl and Eca-109/HADHA cells (D), and the statistical results were shown (E). *P < 0.05, **P < 0.01, ***P < 0.001.

To elucidate the mechanisms underlying HADHA-mediated MDM2 expression, we used the TRRUST database ( https://www.grnpedia.org/trrust) to predict the upstream transcription factors of MDM2 and the STRING database ( https://cn.string-db.org) to identify potential proteins that interact with HADHA. A string of transcription factors, including SP1, was predicted to regulate MDM2, and a STRING gene interaction network was generated ( Supplementary Table S2 and Figure 7A). SP1 is a transcription factor that can bind to the promoter of *MDM2* and increase MDM2 expression [16]. The Sp1 transcription factor is the only one linked to HADHA. To confirm the interaction between SP1 and HADHA, total protein extracts of HADHA-overexpressing Eca-109 cells were immunoprecipitated with anti-HADHA and anti-SP1 antibodies, and western blot analysis confirmed that HADHA and SP1 could be co-immunoprecipitated with each other ( Figure 7B). The dual-luciferase reporter assay was performed in Eca-109 cells, and the transfection of *SP1* and *HADHA* increased the reporter activity of *MDM2*, whereas co-transfection generated the strongest activity ( Figure 7C). Furthermore, ChIP analysis of the *MDM2* promoter revealed that SP1 occupancy was significantly increased in HADHA-overexpressing Eca-109 cells ( Figure 7D,E). Collectively, these results indicated that HADHA bound to the transcription factor SP1 and then enhanced the effect of SP1 on *MDM2* transcription, thereby promoting EC proliferation and resulting in poor survival.

## Discussion

The role of HADHA in cancer has seldom been investigated, and previous studies have reported controversial functions of HADHA in several types of cancer. In liver cancer, the oncogene UBE20 ubiquitinates HADHA for degradation and promotes the reprogramming of lipid metabolism, thereby contributing to tumorigenesis and progression [17]. Similarly, HADHA overexpression disrupts lipid metabolism and decreases the number of cytoplasmic lipid droplets, impairing the energy supply in clear cell renal cell carcinoma [13]. In contrast, HADHA inhibition impairs the mitochondrial respiratory chain and reduces lung tumor size in an animal model [12]. Specifically, HADHA expression is associated with the response to platinum-based chemotherapy and is upregulated in cisplatin-resistant lung cancer cells [18]. Accordingly, the function of HADHA may be more complicated than expected and may vary with different genetic backgrounds. In both liver cancer and clear cell renal cell carcinoma, HADHA overexpression leads to reduced lipid accumulation, supporting cancer cell proliferation and metastasis. However, the function of HADHA in the mitochondrial respiratory chain is essential for lung cancer growth, and the loss of HADHA impairs energy production. Although HADHA plays a proliferative role in ECs, we did not investigate its effects on fatty acid metabolism or the mitochondrial respiratory chain. In future studies, investigating how HADHA alterations regulate the energy supply in EC cells will be important.

The mTOR signaling pathway is crucial for multiple cellular processes, including the maintenance of genomic stability, cell metabolism, protein synthesis, and autophagy [ 19‒ 21]. The activated mTOR signaling pathway contributes to different metabolic pathways, including the lipid, nucleotide, and glucose metabolism pathways, all of which are necessary for cell growth and division. In cancer cells, dozens of oncogenic signaling pathways, including the PI3K/Akt and MAPK pathways, lead to hyperactivation of the mTOR signaling pathway. In EC, silencing of the mTOR signaling pathway by siRNA or rapamycin strongly inhibits proliferation and causes cell cycle arrest [22]. Since rapamycin was first identified in 1964, mTOR inhibitors have been investigated for nearly six decades, and several drugs have been approved by the Food and Drug Administration of USA, including temsirolimus in 2007 and everolimus in 2010 [23]. Although numerous mTOR inhibitors have shown promising results *in vitro* and *in vivo*, none have been administered to treat EC in clinical practice. The combined use of mTOR inhibitors with other chemotherapy agents and the selection of a specific group of patients still holds promise for future studies. Since HADHA upregulation activates the mTOR signalling pathway, it is unclear whether mTOR inhibitors can function better in patients with high HADHA expression.

Although the MDM2 and mTOR signaling pathways were increased after HADHA upregulation, their correlation was not investigated in this study. mTOR and MDM2 are downstream of PI3K-Akt signaling. A recent study revealed that an mTOR inhibitor blocked early-onset tumor formation in p53-null mice [24]. Accordingly, mTOR activation may result in MDM2-mediated p53 degradation. Conversely, the mTOR pathway modulates the protein level of MDM2. The activation of mTOR signaling by insulin-like growth factor-1 or hepatocyte growth factor promotes MDM2 translation in a PI3K-AKT-dependent manner, whereas rapamycin inhibits mTORC1 and downregulates MDM2 [ 25, 26]. Therefore, the correlation between the activation of mTOR signaling and MDM2 upregulation could be a dichotomous interaction.

Further studies are necessary to demonstrate the function of HADHA in EC. First, as essential elements in the beta-oxidation of long-chain fatty acids, the levels of lipid metabolism and energy production must be investigated. Since HADHA can activate the mTOR signaling pathway, it will be of interest to determine whether HADHA could be used as a marker of the efficacy of the mTOR inhibitor. However, the correlation between the mTOR signaling markers MDM2 and HADHA in patients with EC was not confirmed in our study. The cell lines used in the study were EC9076, KYSE450, Eca-109 and TE-1, all of which are esophageal squamous carcinoma cell lines. It is not clear whether the results of this study apply to esophageal adenocarcinoma. Moreover, only one type of cell-related change was analyzed in the RNA-seq experiment, and the results might be more robust if RNA-seq was performed in other esophageal cell lines.

In summary, we showed that HADHA expression was upregulated in EC tissues and was positively correlated with more aggressive clinical manifestations, including tumor infiltration, lymphatic metastasis, tumor stage and distant metastasis. Downregulation of HADHA abrogated EC cell proliferation both *in vitro* and *in vivo*, induced apoptosis, caused cell cycle arrest, and suppressed cell migration ability. In addition, HADHA activated the mTOR signaling pathway, and an mTOR inhibitor significantly blocked the oncogenic effect of HADHA. Furthermore, we showed that HADHA bound to SP1 and enhanced MDM2 expression, subsequently facilitating EC progression. These results indicated that HADHA and MDM2 may act as prognostic indicators and potential therapeutic targets for EC treatment.

## Supporting information

24123_Supplementary_figures

24123Supplementary_Table_1

24123Supplementary_Table_2

## Supplementary Data

Supplementary Data is available at *Acta Biochimica et Biophysica Sinica* online.
