# Supplementary material for: HADHA promotes esophageal cancer progression by activating mTOR signaling and the SP1/MDM2 axis: HADHA promotes esophageal cancer progression
Source: Acta Biochim Biophys Sin (Shanghai). 2024 Sep 26;57(3):378–88. doi: 10.3724/abbs.2024139 (PMC11986453; doi:10.3724/abbs.2024139)
Supplement: 24123_Supplementary_figures [file 24123_Supplementary_figures.docx]

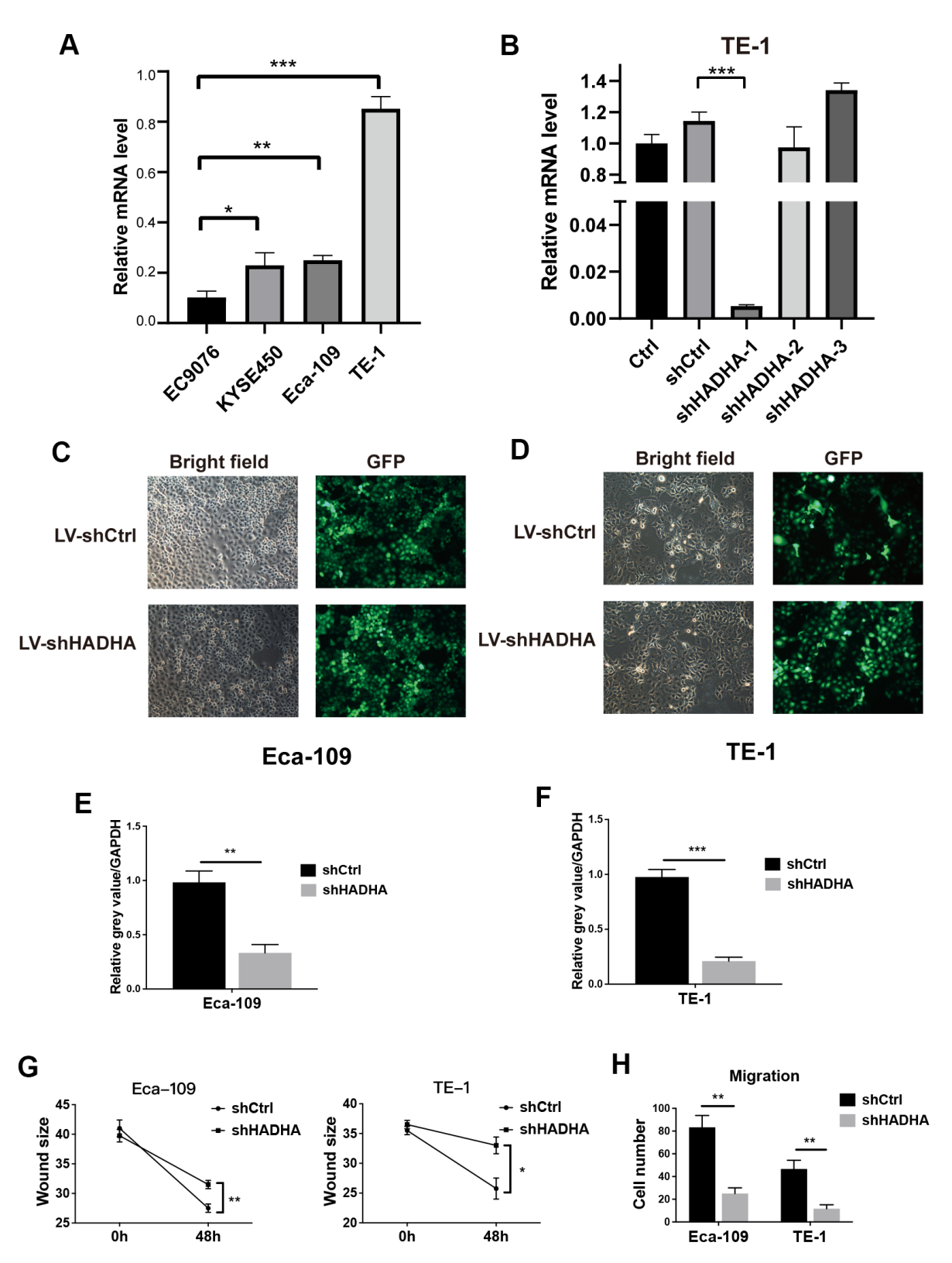


**Supplementary Figure S1. The plasmids were successfully transfected into EC cells** (A) The relative mRNA expression of *HADHA* was examined in EC cell lines. (B) The relative mRNA expression of *HADHA* was examined in TE-1/shHADHA cells. Representative photographs of Eca-109 cells (C) and TE-1 cells (D) transfected with plasmids. The intensity of HADHA expression normalized by negative control in Eca-109 cells (E) and TE-1 cells (F). The relevant statistical graphs of the results in wound healing assay (G) and transwell migration assay (H). Magnification: 100×. **P*<0.05, ***P*<0.01, ****P*<0.001.


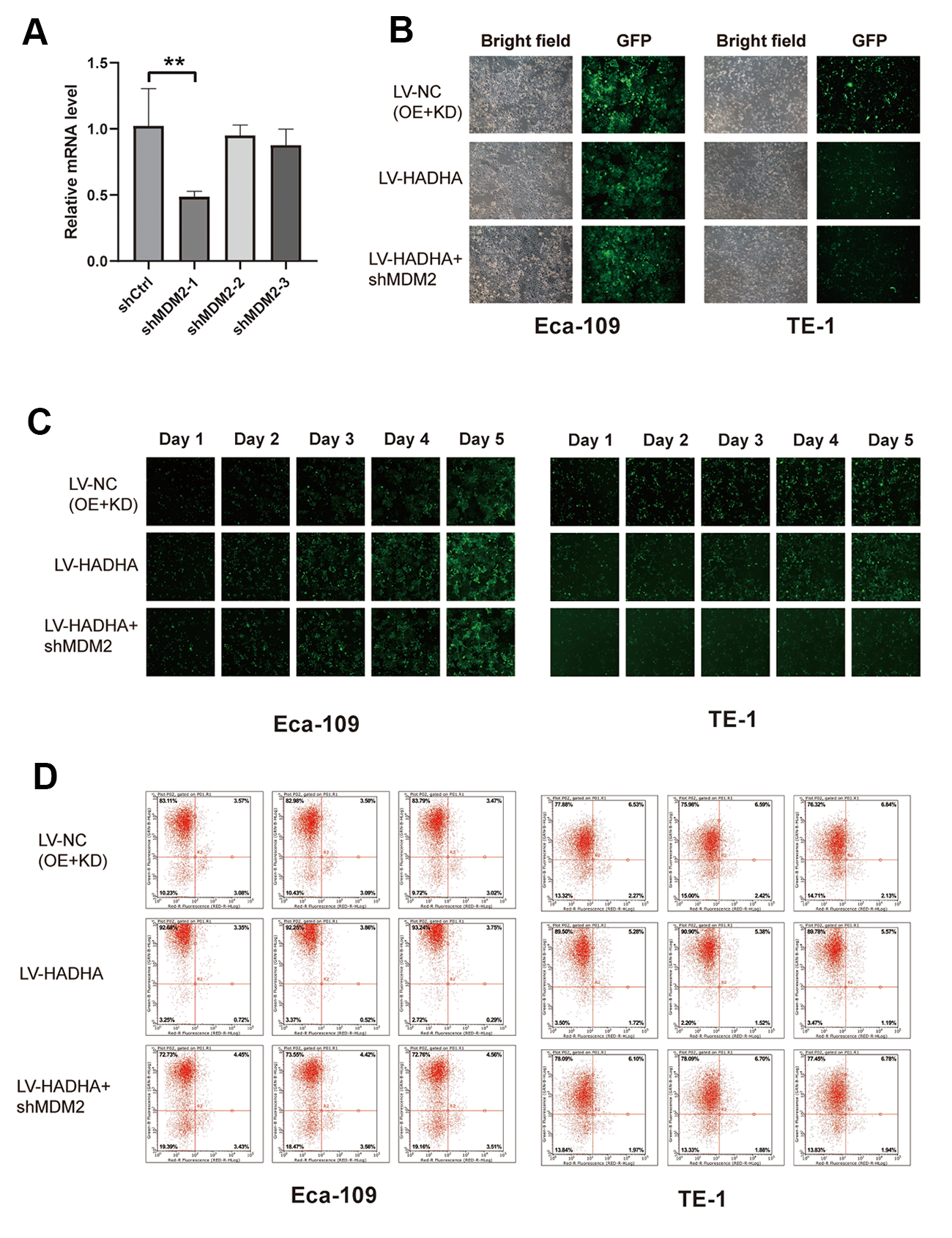


**Supplementary Figure S2. MDM2 participates in HADHA-induced EC progression** (A) The relative mRNA expression of *MDM2* was examined in Eca-109 cells. (B) Representative photographs of Eca-109 cells and TE-1 cells transfected with plasmids. (C) Celigo cell counting assay was performed to assess the abilities of EC cell growth. (D) Representative photographs of apoptosis in different groups of EC cells. Magnification: 100×. ***P*<0.01.
