# Supplementary material for: HADHA promotes esophageal cancer progression by activating mTOR signaling and the SP1/MDM2 axis: HADHA promotes esophageal cancer progression
Source: Acta Biochim Biophys Sin (Shanghai). 2024 Sep 26;57(3):378–88. doi: 10.3724/abbs.2024139 (PMC11986453; doi:10.3724/abbs.2024139)
Supplement: 24123Supplementary_Table_1 [file 24123Supplementary_Table_1.docx]

| Genes | Forward primer sequence (5′→3′) | Reverse prime sequence (5′→3′) |
| --- | --- | --- |
| *GAPDH* | CTGCTGGAGATTATCACGACCG | TGATGACCTTCCCCTGCTTG |
| *HADHA* | TGACTTCAACAGCGACACCCA | CACCCTGTTGCTGTAGCCAAA |
| *IRS1* | GGTGGATGACTCTGTGGTGG | GGACGCTGATGGGGTTAGAG |
| *PIK3CB* | CTGCGACAGATGAGTGATGAAG | CCCTATCCTCCGATTACCAAG |
| *MDM2* | GGTGAGGAGCAGGCAAATGT | CGAAGCTGGAATCTGTGAGGT |
| *PPP2CA* | GTTCCCCATGAGGGTCCAAT | TTGCCCAAAGGTGTAACCAG |
| *EIF4EBP1* | ACTCACCTGTGACCAAAACACC | CCGCTTATCTTCTGGGCTATT |
| *PIK3R1* | GAAGAAATTGGCTGGTTAAATGG | CTGGTGCAACAGGAAGAGGC |
| *RALB* | ACTGCTCGTCGTGGGAAACA | CCACTCTTCGGCTTTACTCCTG |
| *RAP2B* | CTATTTGCCGAGATCGTGCG | AGAGAGTCGGATGCGTTTGG |

**Supplementary Table S1. The sequences of primers used in the study**
