# Supplementary material for: HADHA promotes esophageal cancer progression by activating mTOR signaling and the SP1/MDM2 axis: HADHA promotes esophageal cancer progression
Source: Acta Biochim Biophys Sin (Shanghai). 2024 Sep 26;57(3):378–88. doi: 10.3724/abbs.2024139 (PMC11986453; doi:10.3724/abbs.2024139)
Supplement: 24123Supplementary_Table_2 [file 24123Supplementary_Table_2.docx]

| Transcription factors | Target | Regulation type | Reference (PMID) |
| --- | --- | --- | --- |
| BRCA1 | MDM2 | Activation | 11256609 |
| E2F1 | MDM2 | Repression | 20837136 |
| ELF4 | MDM2 | Unknown | 23393136 |
| ELL | MDM2 | Repression | 15851483 |
| EP300 | MDM2 | Unknown | 11591713 |
| ESR1 | MDM2 | Unknown | 12897156 |
| ETS1 | MDM2 | Repression | 11424091 |
| ETS2 | MDM2 | Activation | 12750288 |
| FOSL2 | MDM2 | Activation | 22493372 |
| FUS | MDM2 | Repression | 17234782 |
| JUND | MDM2 | Activation | 22493372 |
| JUND | MDM2 | Repression | 18071306 |
| MLLT10 | MDM2 | Repression | 15851483 |
| MLLT3 | MDM2 | Repression | 15851483 |
| MYCN | MDM2 | Unknown | 15644444;15927364 |
| NFATC2 | MDM2 | Activation | 22787160 |
| NPM1 | MDM2 | Repression | 23393136 |
| NR4A1 | MDM2 | Repression | 17139261 |
| SKI | MDM2 | Activation | 22411991 |
| SP1 | MDM2 | Activation | 15550242;17214373;17634539 |
| SP1 | MDM2 | Unknown | 18026875;23584477 |
| TFAP4 | MDM2 | Repression | 19505873 |
| TP53 | MDM2 | Activation | 10566557;10718212;12902982;15851483;20837136;22223137;22532570;9645455;9858609 |
| TP53 | MDM2 | Unknown | 10469568;12851404;14532965;15856024;18566224;18583933;20036872;22180176;9071998 |
| TP73 | MDM2 | Unknown | 10469568 |
| ZBTB2 | MDM2 | Activation | 19380588 |

**Supplementary Table S2. Transcription factors predicted to regulate MDM2 by the TRRUST database**
